# Supplementary material for: Deciphering the contributing motifs of reconstructed cobalt (II) sulfides catalysts in Li-CO2 batteries
Source: Nat Commun. 2024 Mar 9;15:2167. doi: 10.1038/s41467-024-46465-8 (PMC10924882; doi:10.1038/s41467-024-46465-8)
Supplement: Supplementary file 1 — Supplementary Information [file 41467_2024_46465_MOESM1_ESM.pdf]

Supplementary information for

Deciphering the Contributing Motifs of Reconstructed Cobalt (II) Sulfides Catalysts in Li-CO_2 Batteries

Yingqi Liu[1,†], Zhiyuan Zhang[1,†], Junyang Tan[1], Biao Chen[2], Bingyi Lu[1], Rui Mao[1], Bilu Liu[1], Dashuai Wang[3,*], Guangmin Zhou[1,*], Hui-Ming Cheng [4,5,6,*]

1 Tsinghua-Berkeley Shenzhen Institute & Tsinghua Shenzhen International Graduate School, Tsinghua University, Shenzhen, 518055, People's Republic of China

2 School of Materials Science and Engineering and Tianjin Key Laboratory of Composite and Functional Materials, Tianjin University, Tianjin, 300350, People's Republic of China

3 Institute of Zhejiang University-Quzhou & Key Laboratory of Biomass Chemical Engineering of Ministry of Education, College of Chemical and Biological Engineering, Zhejiang University, Hangzhou, 310027, China

4 Shenyang National Laboratory for Materials Science, Institute of Metal Research, Chinese Academy of Sciences, Shenyang 110016, China

5 Institute of Technology for Carbon Neutrality, Shenzhen Institute of Advanced Technology, Chinese Academy of Sciences, Shenzhen, 518055, People's Republic of China

6 Shenzhen University of Advanced Technology, Shenzhen 518055, China

E-mail: dswang@zju.edu.cn, guangminzhou@sz.tsinghua.edu.cn, hm.cheng@siat.ac.cn, cheng@imr.ac.cn

[†] These authors were equal major contributors.

This file includes:

Supplementary Figures S1 to S36

Supplementary Tables S1 to S11

Supplementary Reference

Supplementary Figures

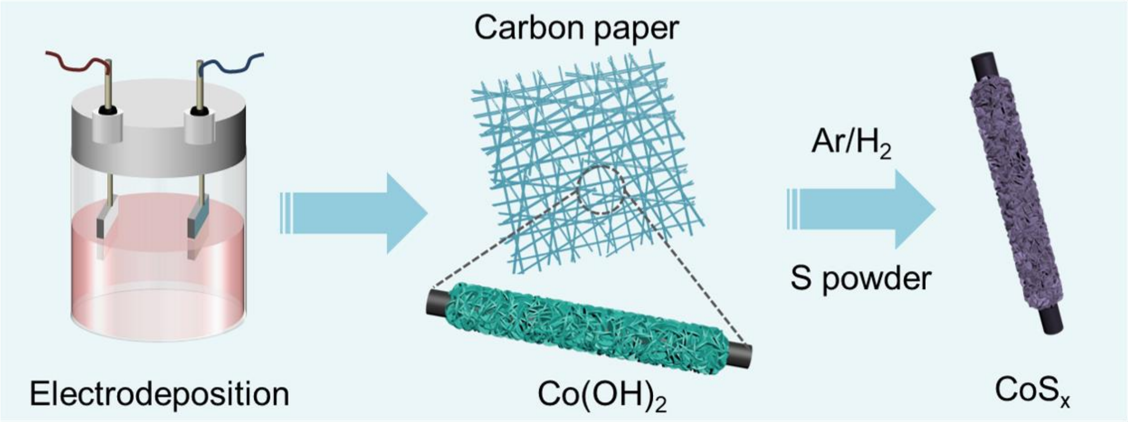

Supplementary Fig. 1. Schematic of the synthesis process of cobalt sulfides.

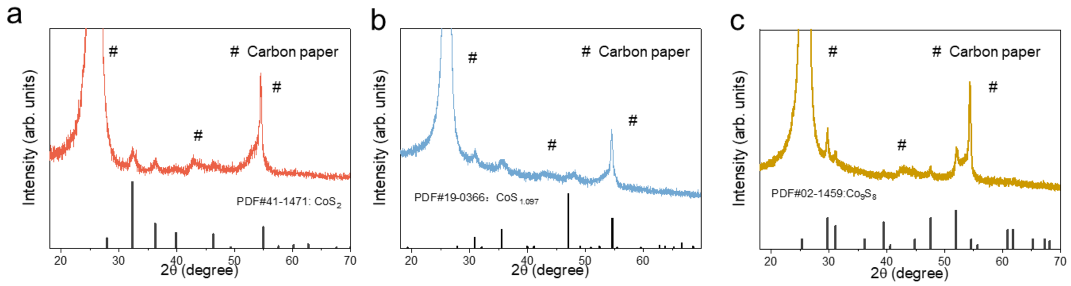

Supplementary Fig.2.XRD patterns of CoS_x. a CoS_2, **b** $CoS_{1.097}$, and **c** Co_9S_8.

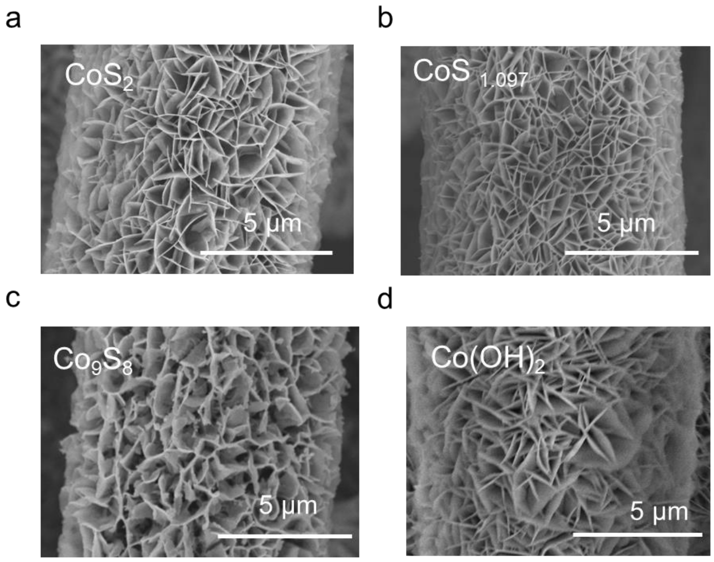

Supplementary Fig.3. SEM images of CoS_x and precursor. a CoS_2, **b** CoS $_{1.097}$, **c** Co_9S_8, and **d** $Co(OH)_2$.

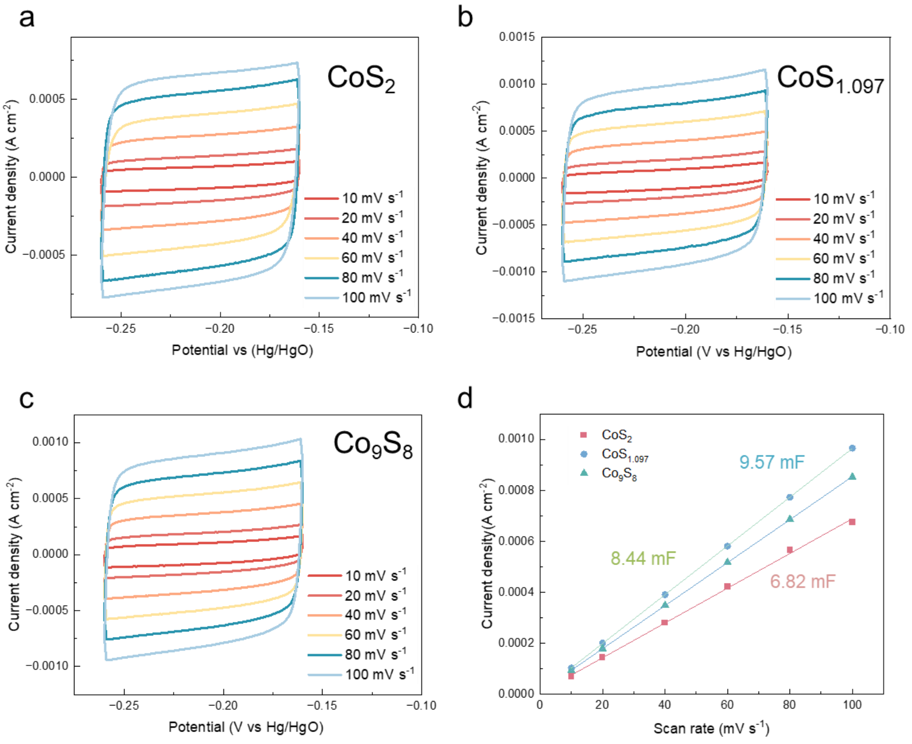

Supplementary Fig.4 ECSA test results. CV curves of **a** CoS_2, **b** $CoS_{1.097}$, and **c** Co_9S_8. **d** C_{dl} of the three cathodes.

ECSA are determined by C_{dl} based on CV scans in no non-Faradaic region as shown in Supplementary Fig. 4a-c. The values are calculated in Supplementary Fig.4d, which of CoS_2, $CoS_{1.097}$, and Co_9S_8 are 6.82, 9.75, and 8.44 mF cm^{-2}.

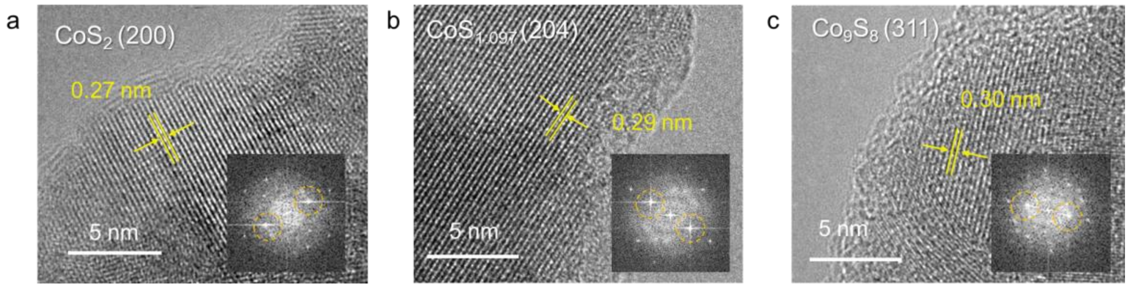

Supplementary Fig.5. TEM images. a CoS_2, **b** $CoS_{1.097}$, and **c** Co_9S_8. The insets are the corresponding Fast Fourier Transform (FFT) patterns.

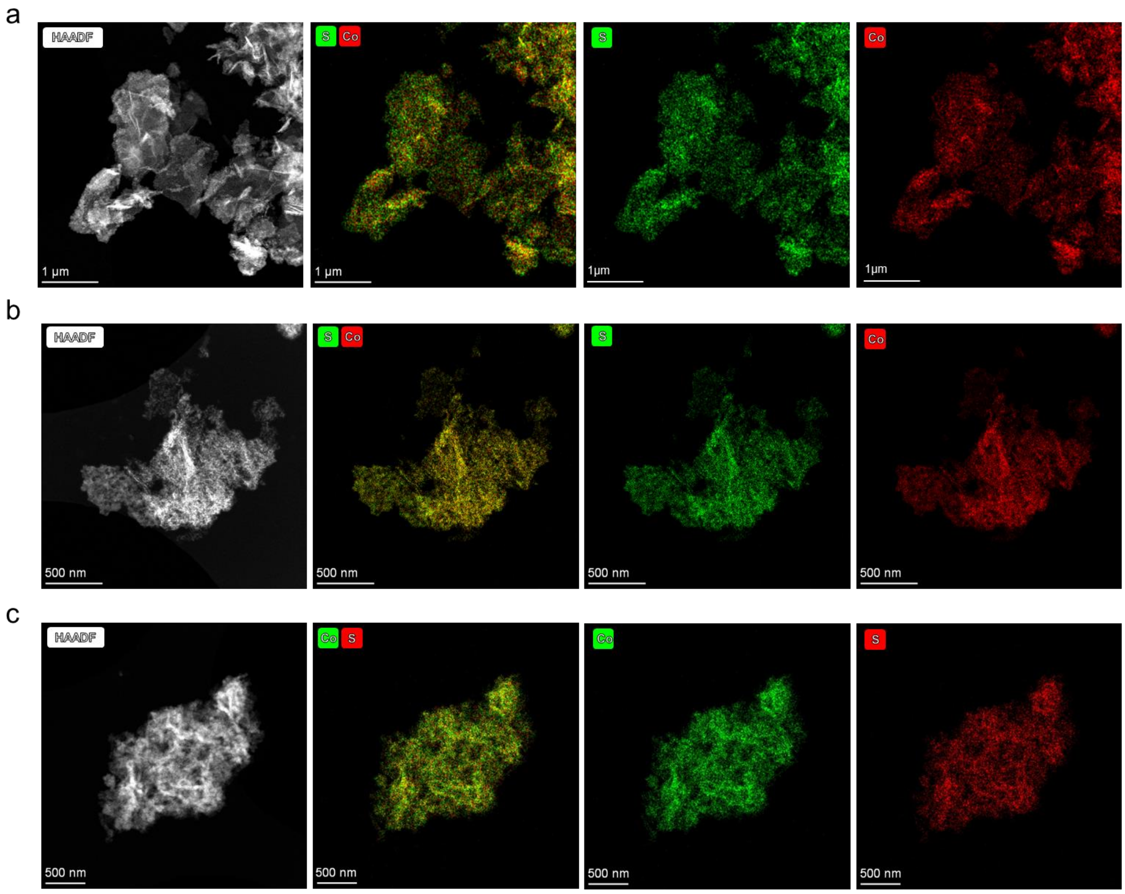

Supplementary Fig.6. HAADF-STEM images and EDS mappings. a CoS_2, **b** $CoS_{1.097}$, and **c** Co_9S_8.

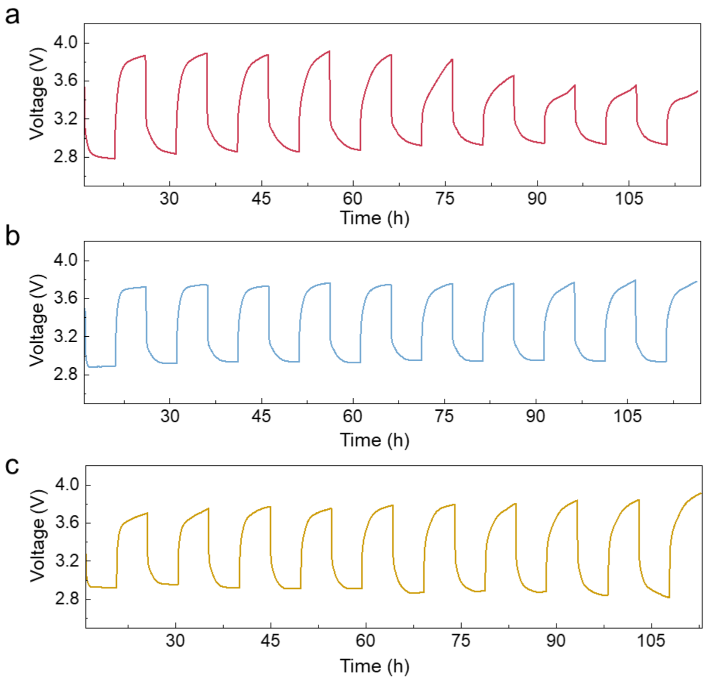

Supplementary Fig.7. Time-voltage curves at 20 μA cm^{-2}. a CoS_2, **b** $CoS_{1.097}$, and **c** Co_9S_8.

In Supplementary Fig.7, we observed that the charge voltage of cobalt sulfides changed from the 5th cycle and became stable in 10 cycles. Therefore, chemical states of the three sulfides after 5 and 10 cycles are selected to study in the latter discussion.

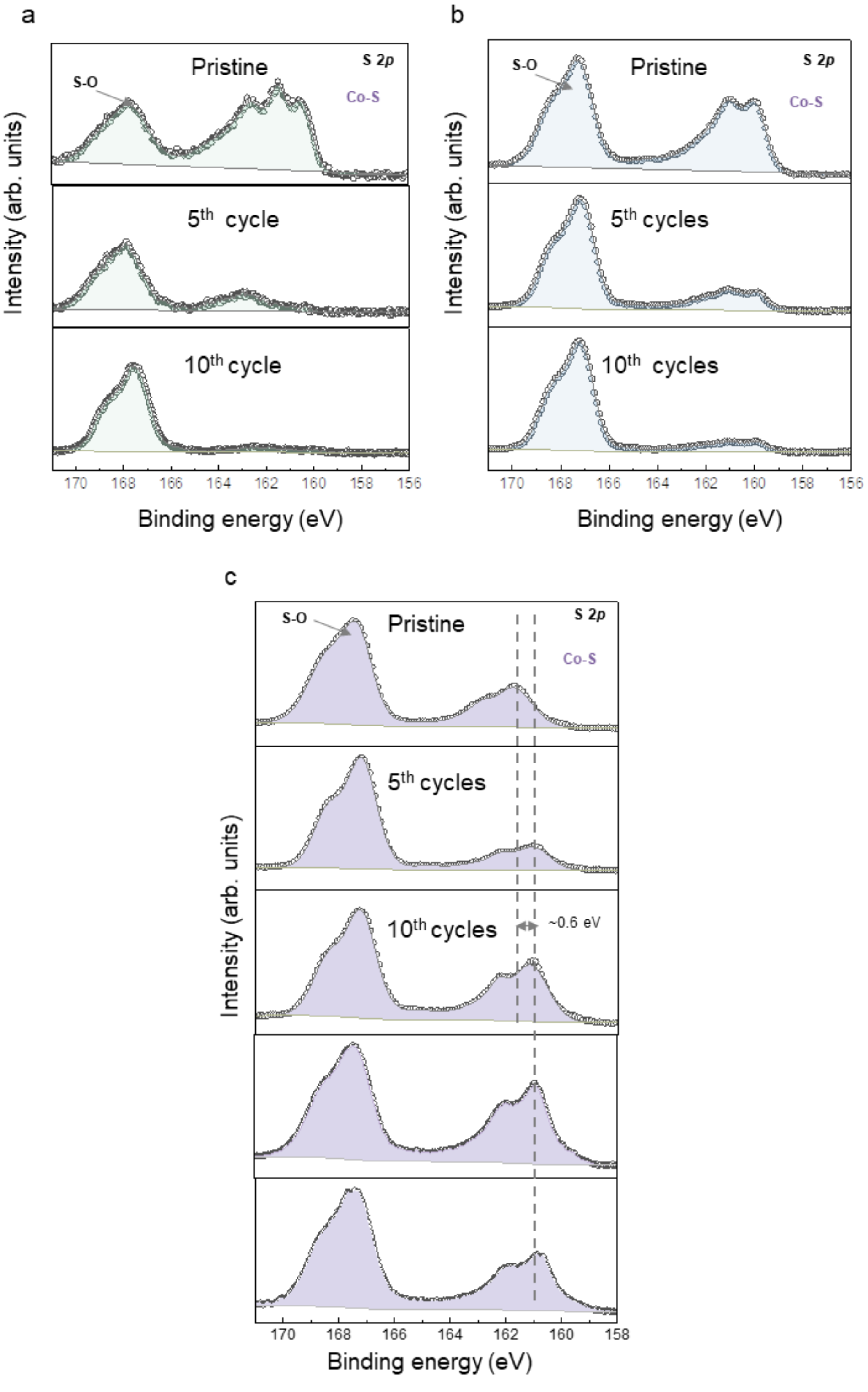

Supplementary Fig.8. XPS results. S 2*p* of **a** Co_9S_8, **b** $CoS_{1.097}$, and **c** CoS_2 cathode for pristine, and after cycling.

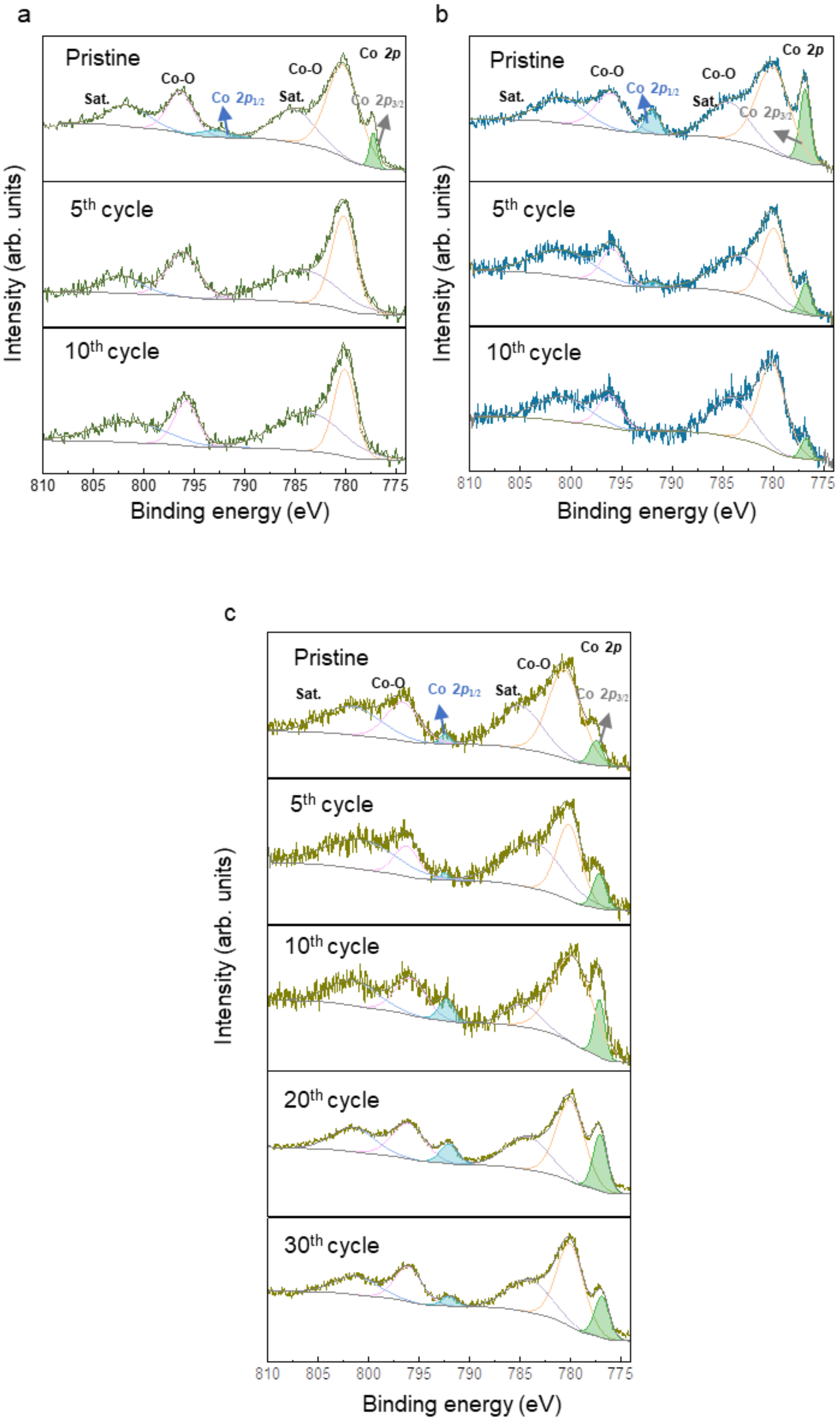

Supplementary Fig.9. XPS results. Co 2*p* of **a** Co_9S_8, **b** $CoS_{1.097}$, and **c** CoS_2 cathode for pristine, and after cycling.

As shown in Supplementary Fig.8, all the high-resolution XPS spectra of the S 2*p* show two pairs of peaks. The peaks in the range of 168~170 eV correspond to

sulfide oxide due to the susceptibility of sulfides to oxidation in air, in accordance with prior reports.[1, 2] The peaks at lower binding energy can be fitted into two peaks, corresponding to S 2$p_{1/2}$ and S 2$p_{3/2}$.[3, 4, 5] The intensity of the S 2$p_{1/2}$ and S 2$p_{3/2}$ peaks of $CoS_{1.097}$ and Co_9S_8 decreases after 5 and 10 cycles without notable location changes. These findings are consistent with the decline in the intensity of the Co-S binding peaks at 777.4 eV and 777.2 eV of $CoS_{1.097}$ and Co_9S_8 in the Co 2*p* spectra after cycling, and that of Co_9S_8 even disappear after 10 cycles (Supplementary Fig.9). These results indicate that cycling causes significant structural changes in both $CoS_{1.097}$ and Co_9S_8.

CoS_2, on the other hand, exhibits no significant decline in the intensity of S 2$p_{1/2}$ and S 2$p_{3/2}$ peaks. Instead, the peaks shift ~0.6 eV to lower binding energy after 5 cycles, without moving after 10, 20, and 30 cycles. These results indicate that S with less than the full coordination is in CoS_2, and the structure of reconstructed CoS_2 remains stable during battery operation.[3, 6]

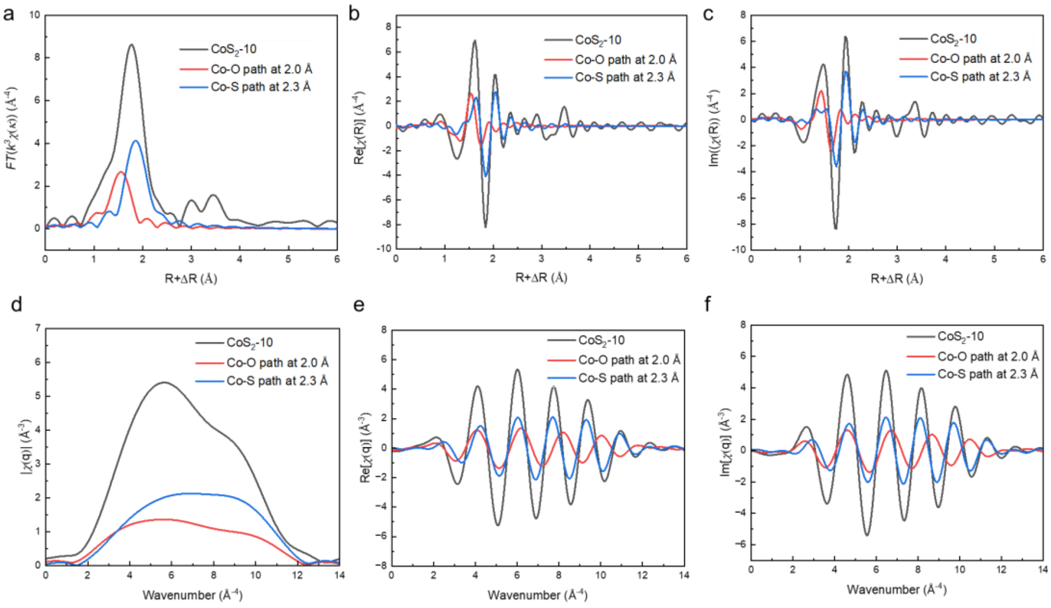

Supplementary Fig.10. Comparison between Co *K*-edge EXAFS of CoS_2-10 with FEFF-calculated Co-O and Co-S path. a magnitude, **b** real component, and **c**, imaginary component of Fourier transformed EXAFS. **d** magnitude, **e** real component, and **f** imaginary component of inverse Fourier transformed EXAFS.

The inverse Fourier transformation was performed in the R-space between 1 and 2.547 Å The Co-O and Co-S path was calculated by ab-intio code FEFF 6[7].

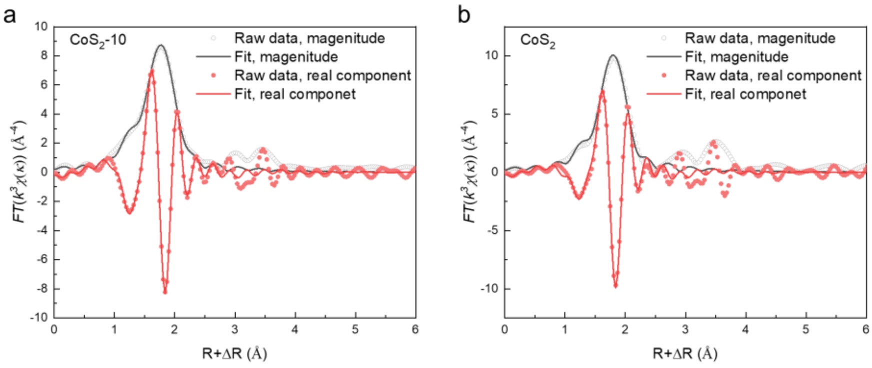

Supplementary Fig.11. The least-squares non-linearly fitting of EXAFS of Co *K*-edge. a CoS_2-10 and **b** CoS_2. The real components of FT-EXAFS are also shown in the figure.

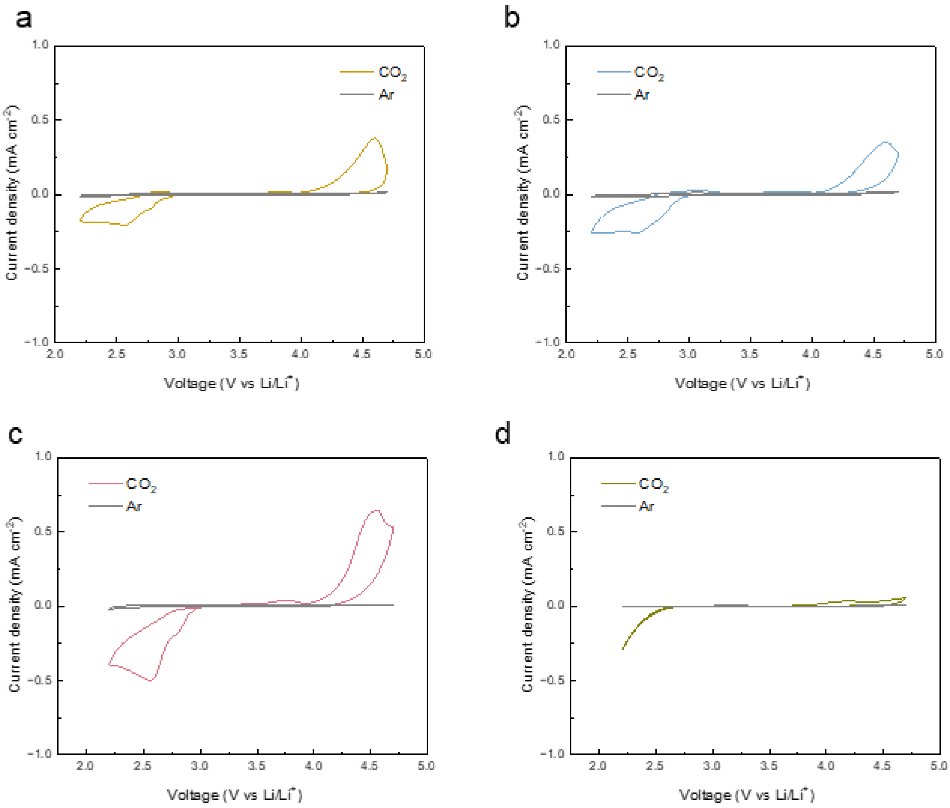

Supplementary Fig.12.CV curves in CO_2 and Ar atmosphere. a Co_9S_8, **b** $CoS_{1.097}$, **c** CoS_2, and **d** Carbon paper.

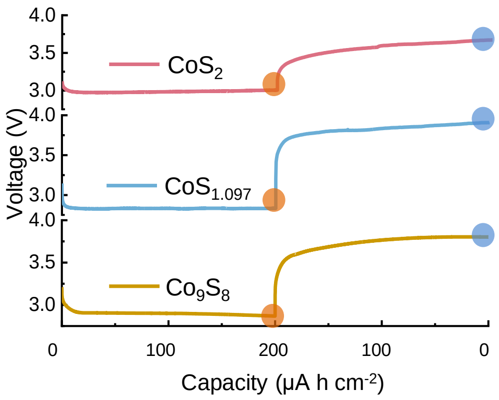

Supplementary Fig.13. Discharge and charge curves of the three cathodes at a current density of 20 μA cm^{-2} with a limited capacity of 200 μA h cm^{-2}.

For the qualitative characterizations, including SEM, XRD and Raman, the capacity is twice than capacity used in the electrochemical tests to obtain more discharge products. Since only one platform during discharge and charge as the capacity increases, the reaction is the same as that with a limited capacity of 200 μA h cm^{-2}.

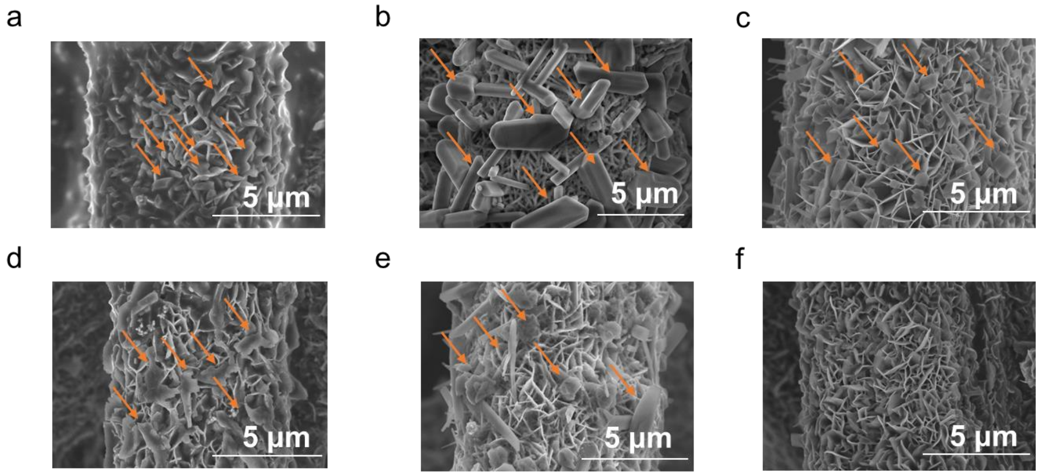

Supplementary Fig.14. SEM images after discharge(upper) and recharge (down) with a limited capacity of 200 μA h cm^{-2}. a,d Co_9S_8, **b,e** $CoS_{1.097,}$ and **c,f** CoS_2.

As the pristine morphologies for all the sulfides are thin flakes vertically on carbon fibers as shown in Supplementary Fig.3, varisized particles on the cathodes are considered as reaction products after discharge and charge. The arrows are added to point out the products formed in batteries.

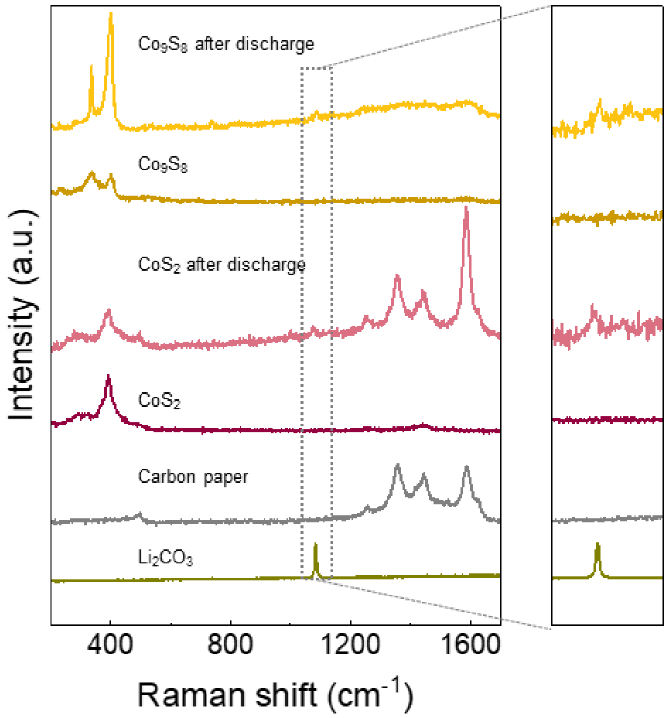

Supplementary Fig.15. Raman spectra of cathodes before and after discharge, and the reference spectrum for Li_2CO_3 as well as carbon paper.

The peaks of products on CoS_2 and Co_9S_8 are not unambiguous to be identified in XRD patterns in Fig. 3b. Thereby, the Raman spectra of the two cathodes before and after discharge are added here. The emerged peaks on CoS_2 and Co_9S_8 after discharge are around 1080 cm^{-1} can be assigned as Li_2CO_3.

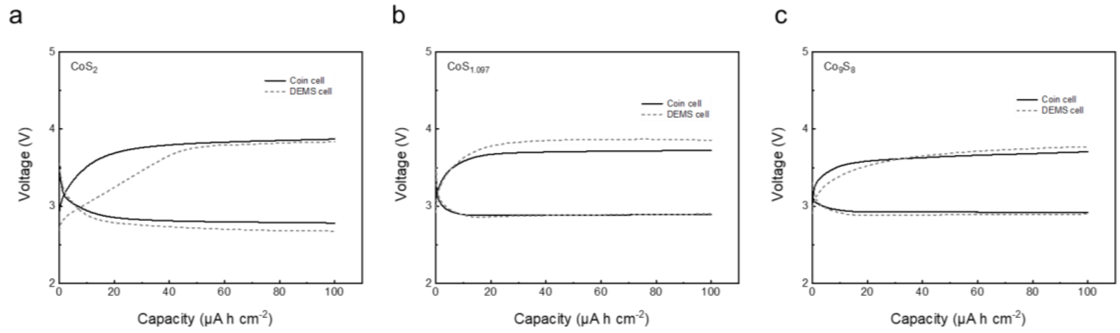

Supplementary Fig.16. The charge/discharge profiles for the 1st cycle for the normal electrochemical cells and the DEMS cells at 20 µA cm^{-2} with a limited capacity of 100 µA h cm^{-2}. a CoS_2, **b** $CoS_{1.097}$, **c** Co_9S_8.

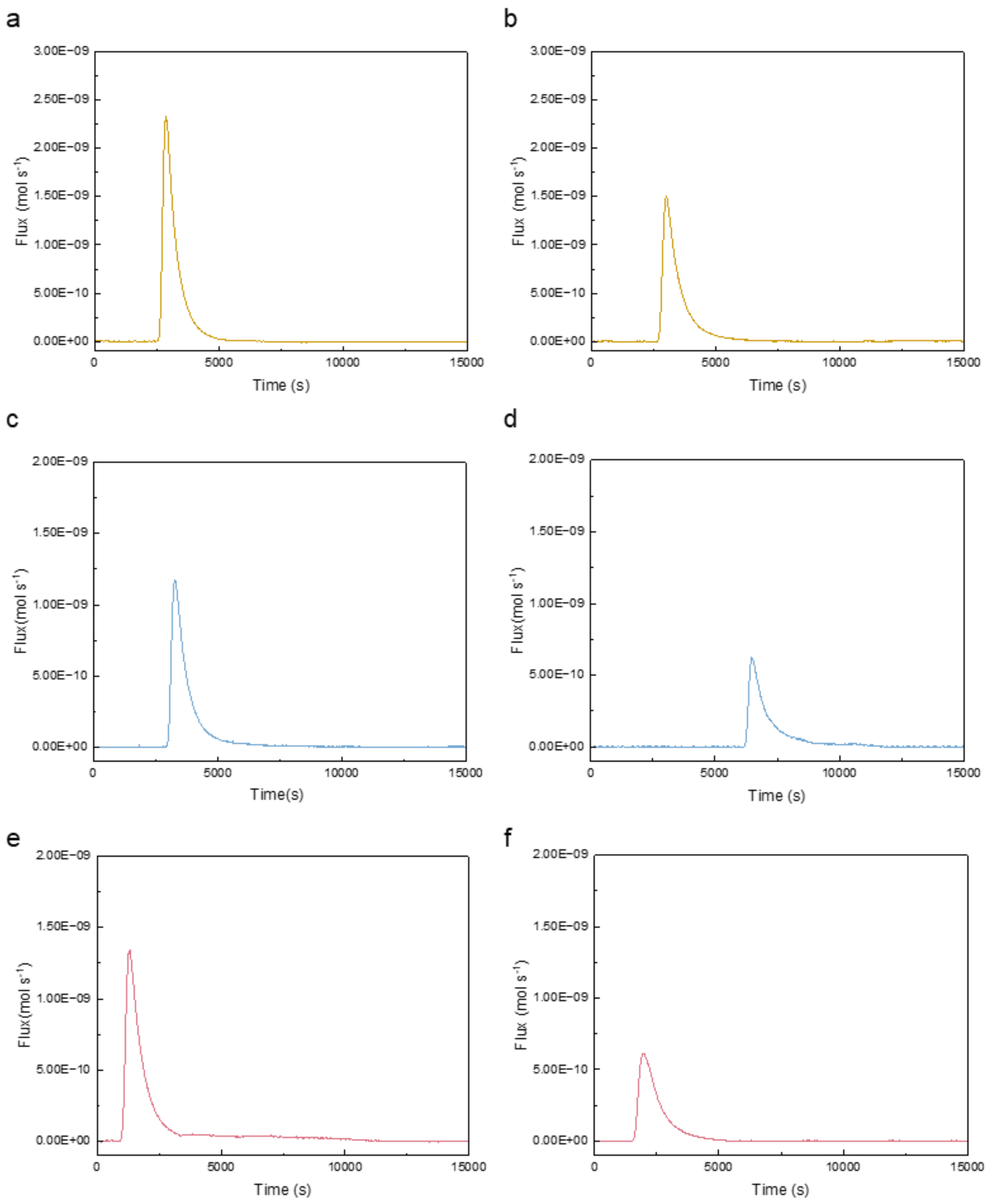

Supplementary Fig.17. DEMS results for titrating discharged and charged cathodes. The CO_2 (m/z=44) signals after titrating **a** discharged and **b** charged Co_9S_8; **c** discharged and **d** charged $CoS_{1.097}$; **e** discharged and **f** charged CoS_2. The current density of discharge and charge is 20 μA cm^{-2} with a limited capacity of 100 μA h cm^{-2}.

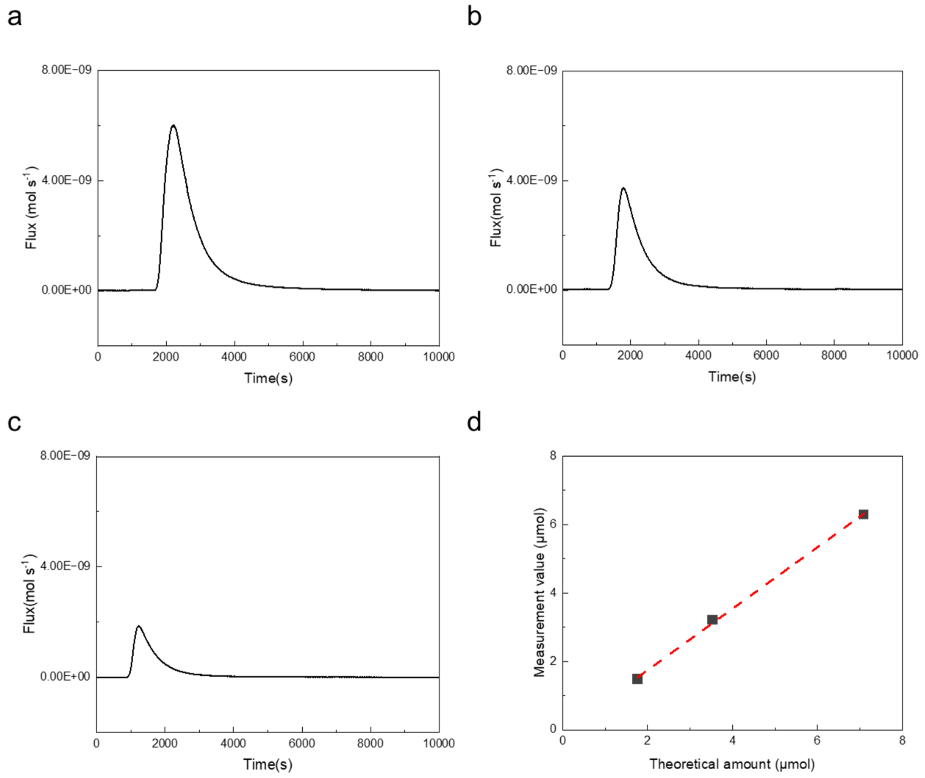

Supplementary Fig.18. DEMS results for titrating Li_2CO_3 solution. The CO_2 (m/z=44) signals after titrating **a** 0.1 mL, **b** 0.05 mL, and **c** 0.025 mL Li_2CO_3 solution with a certain concentration of 5.25 mg mL^{-1}. **d** The relationship between measurement value of CO_2 and theoretical amount of Li_2CO_3.

Titrating Li_2CO_3 solution with a certain concentration is performed as an external standard to diminish the measurement error as shown in Supplementary Fig.18a-c. Supplementary Fig.18d shows that the measurement value is approximately linear with the theoretical amount as a function of y= 0.89847*x-0.05068. y is the measurement value of CO_2 evolution and x is the theoretical amount of Li_2CO_3. The linear relationship is defined as external standard 1#, corresponding to Supplementary Fig.17.

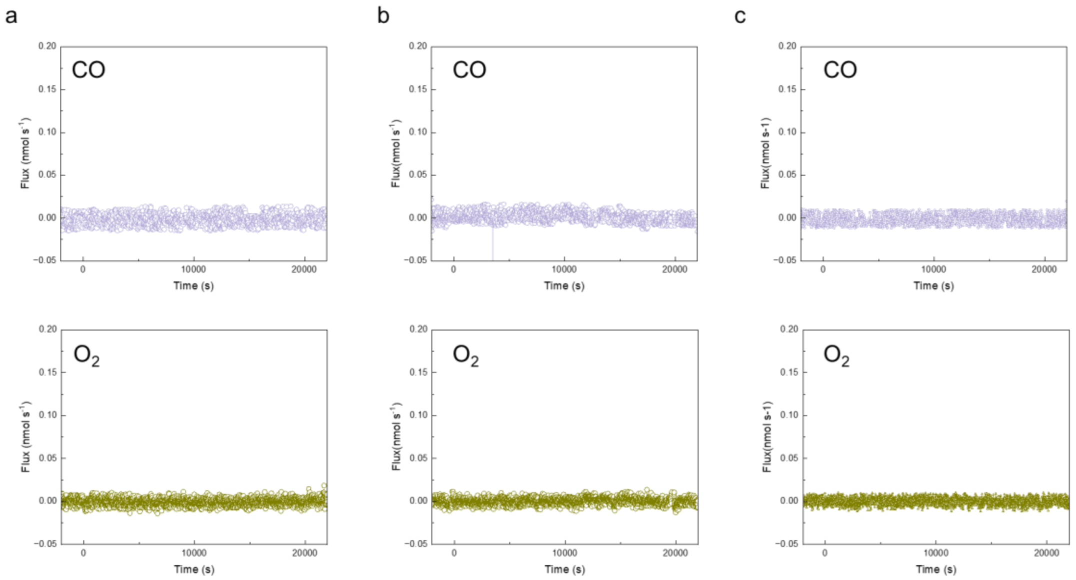

Supplementary Fig.19. The gas (CO and O_2) generation during the charge. a Co_9S_8, **b** $CoS_{1.097}$, and **c** CoS_2.

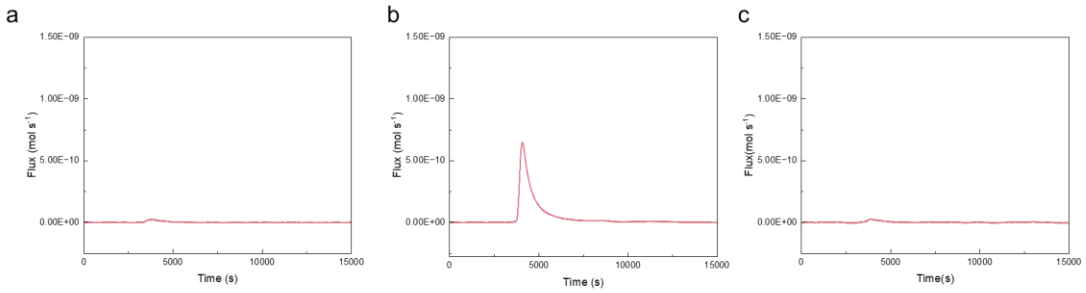

Supplementary Fig.20. DEMS results for titrating cycled cathodes. The CO_2 (m/z=44) generation after titrating CoS_2 cathodes after the **a** 9th charge, **b** 10th discharge, and **c** 10th charge.

The measurement values of CO_2 are 0.023, 0.58 and 0.027 μmol for CoS_2 cathodes after the 9th charge, 10th discharge, and 10th charge.

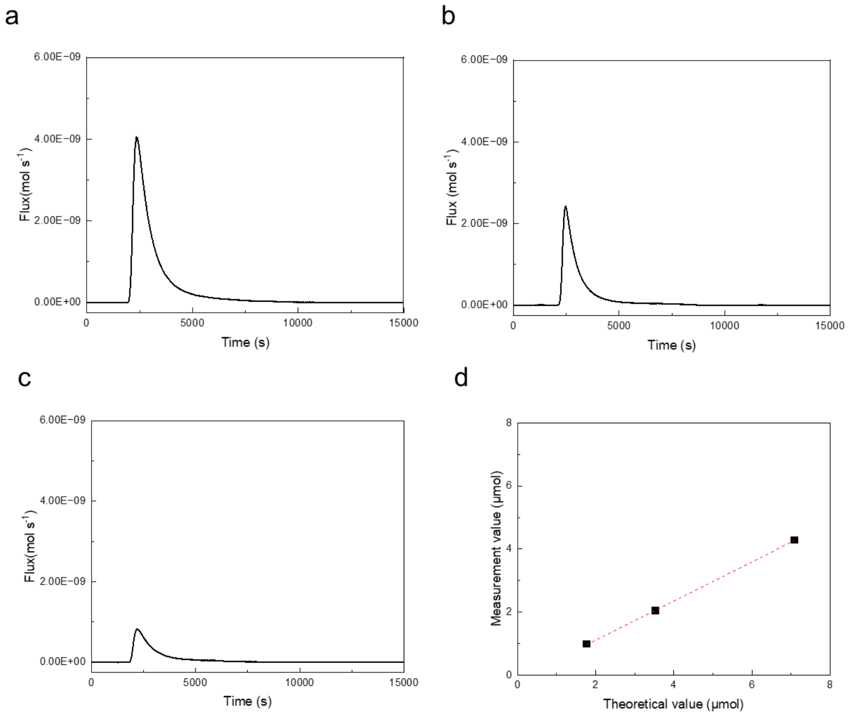

Supplementary Fig.21. DEMS results for titrating Li_2CO_3 solution. The CO_2 (m/z=44) generation after titrating **a** 0.1 mL, **b** 0.05 mL, and **c** 0.025 mL Li_2CO_3 solution with a certain concentration of 5.25 mg mL^{-1}. **d** The relationship between measurement value of CO_2 and theoretical amount of Li_2CO_3.

The corresponding linear relationship between measurement and theoretical results is a function of y=0.6216*x-0.13386 in Supplementary Fig.21d, of which y is the measurement value of CO_2 evolution and x is the theoretical amount of Li_2CO_3. The linear relationship is defined as external standard 2# corresponding to titration experiment after cycling in Supplementary Fig.20.

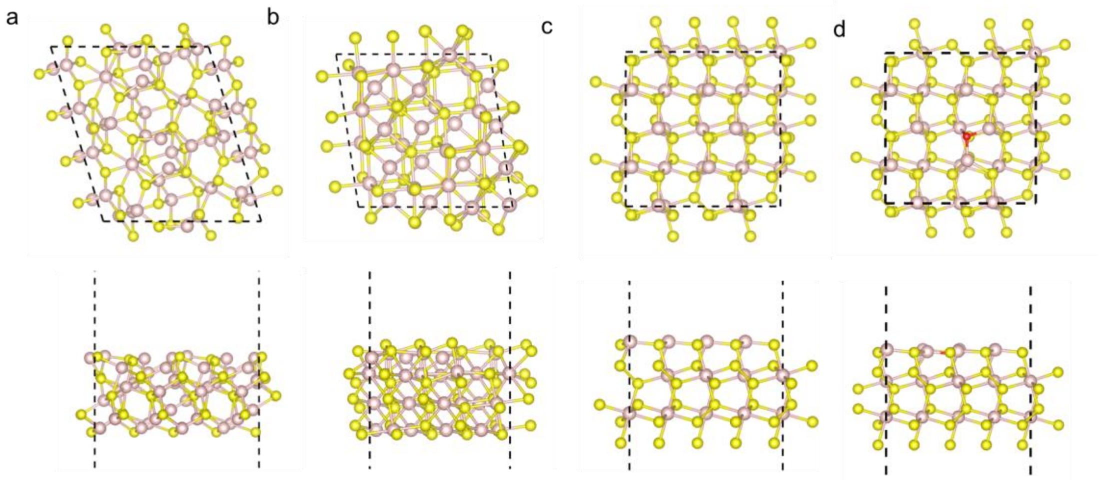

Supplementary Fig.22. Top (up) and side (down) views of constructed CoS_x. a the (311) plane of Co_9S_8, **b** the (204) plane of $CoS_{1.097}$, **c** the (200) plane of CoS_2 and **d** O-CoS_2. (Pink atom: Co; Yellow atom: S; Red atom: O; Green atom: Li; Brown atom: C, which are the same in this paper.)

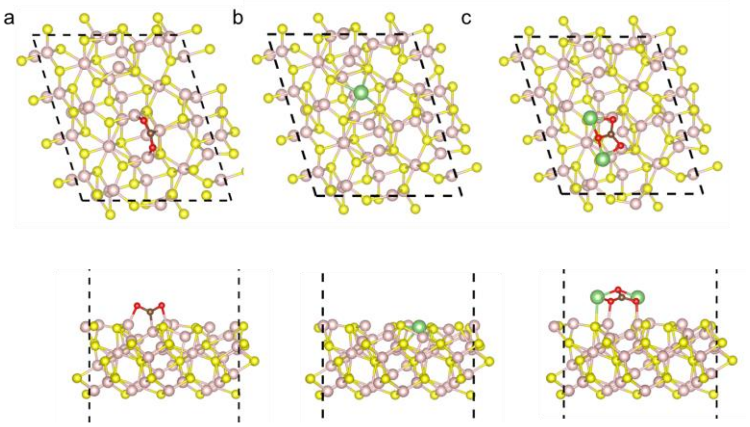

Supplementary Fig.23. Top (up) and side (down) views of adsorptions on Co_9S_8. The **a** CO_2 adsorption configurations, **b** Li adsorption configurations, and **c** Li_2CO_3 adsorption configurations on Co_9S_8.

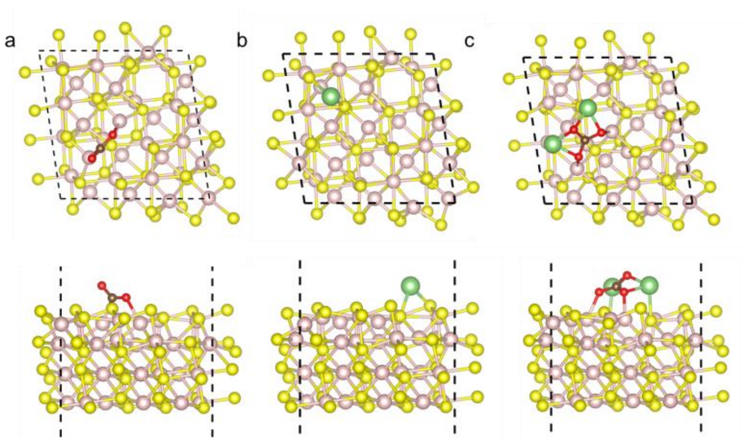

Supplementary Fig.24. Top (up) and side (down) views of adsorptions on $CoS_{1.097}$. The **a** CO_2 adsorption configurations, **b** Li adsorption configurations, and **c** Li_2CO_3 adsorption configurations on $CoS_{1.097}$.

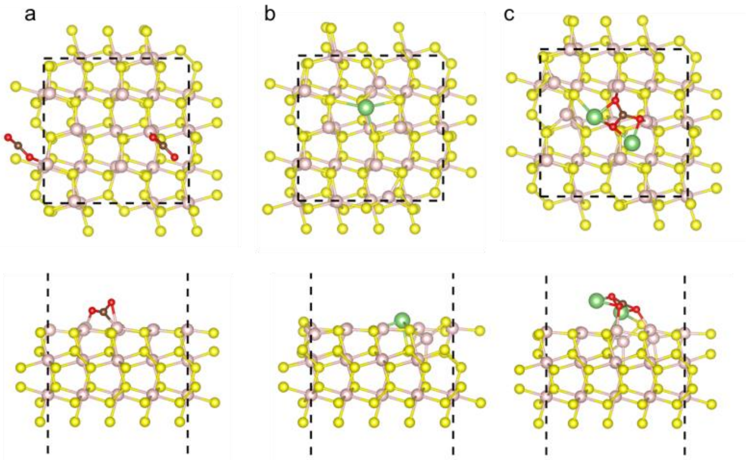

Supplementary Fig.25. Top (up) and side (down) views of adsorptions on CoS_2. The **a** CO_2 adsorption configurations, **b** Li adsorption configurations, and **c** Li_2CO_3 adsorption configurations on CoS_2.

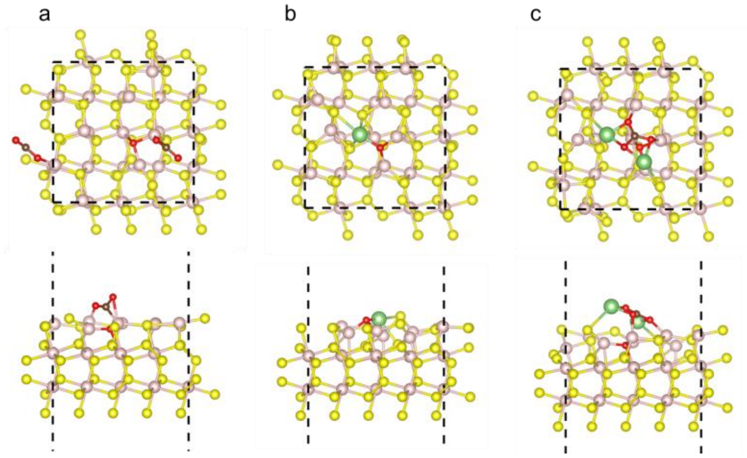

Supplementary Fig.26. Top (up) and side (down) views of adsorptions on O-CoS$_2$. The **a** CO_2 adsorption configurations, **b** Li adsorption configurations, and **c** Li_2CO_3 adsorption configurations on O-CoS$_2$.

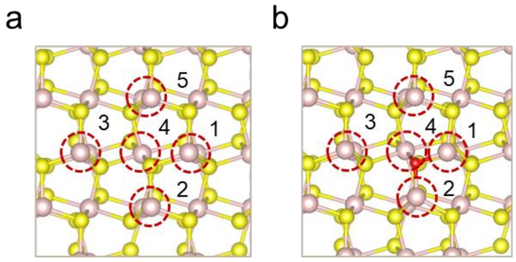

Supplementary Fig.27. Scheme of Co sites 1-5 on CoS$_x$. **a** CoS$_2$ and **b** O-CoS$_2$.

(1) (2) (3) (4) (5) (6) (7) (8)

$$\text{I}^* \xrightarrow{Li} {}^*\text{Li} \xrightarrow{CO_2} {}^*\text{LiCO}_2 \xrightarrow{Li} {}^*\text{Li}_2\text{CO}_2 \xrightarrow{CO_2} {}^*\text{Li}_2\text{CO}_3 + {}^*\text{CO} \rightarrow {}^*\text{CO} + \text{Li}_2\text{CO}_3 \xrightarrow{CO_2} {}^*\text{CO}_3 + {}^*\text{C} \xrightarrow{Li} {}^*\text{LiCO}_3 + {}^*\text{C} \xrightarrow{Li} {}^*\text{Li}_2\text{CO}_3 + {}^*\text{C}$$

$$\text{II}^* \xrightarrow{Li} {}^*\text{Li} \xrightarrow{CO_2} {}^*\text{LiCO}_2 \xrightarrow{CO_2} {}^*\text{LiCO}_3 + {}^*\text{CO} \xrightarrow{Li} {}^*\text{Li}_2\text{CO}_3 + {}^*\text{CO} \rightarrow {}^*\text{CO} + \text{Li}_2\text{CO}_3 \xrightarrow{CO_2} {}^*\text{CO}_3 + {}^*\text{C} \xrightarrow{Li} {}^*\text{LiCO}_3 + {}^*\text{C} \xrightarrow{Li} {}^*\text{Li}_2\text{CO}_3 + {}^*\text{C}$$

$$\text{III}^* \xrightarrow{CO_2} {}^*\text{CO}_2 \xrightarrow{Li} {}^*\text{LiCO}_2 \xrightarrow{CO_2} \text{LiCO}_{3+}{}^*\text{CO} \xrightarrow{Li} {}^*\text{Li}_2\text{CO}_3 + {}^*\text{CO} \rightarrow {}^*\text{CO} + \text{Li}_2\text{CO}_3 \xrightarrow{CO_2} {}^*\text{CO}_3 + {}^*\text{C} \xrightarrow{Li} {}^*\text{LiCO}_3 + {}^*\text{C} \xrightarrow{Li} {}^*\text{Li}_2\text{CO}_3 + {}^*\text{C}$$

$$\text{IV}^* \xrightarrow{CO_2} {}^*\text{CO}_2 \xrightarrow{Li} {}^*\text{LiCO}_2 \xrightarrow{Li} {}^*\text{Li}_2\text{CO}_2 \xrightarrow{CO_2} {}^*\text{Li}_2\text{CO}_3 + {}^*\text{CO} \rightarrow {}^*\text{CO} + \text{Li}_2\text{CO}_3 \xrightarrow{CO_2} {}^*\text{CO}_3 + {}^*\text{C} \xrightarrow{Li} {}^*\text{LiCO}_3 + {}^*\text{C} \xrightarrow{Li} {}^*\text{Li}_2\text{CO}_3 + {}^*\text{C}$$

$$\text{V}^* \xrightarrow{CO_2} {}^*\text{CO}_2 \xrightarrow{CO_2} {}^*\text{CO}_3 + {}^*\text{CO} \xrightarrow{Li} {}^*\text{LiCO}_3 + {}^*\text{CO} \xrightarrow{Li} {}^*\text{Li}_2\text{CO}_3 + {}^*\text{CO} \rightarrow {}^*\text{CO} + \text{Li}_2\text{CO}_3 \xrightarrow{CO_2} {}^*\text{CO}_3 + {}^*\text{C} \xrightarrow{Li} {}^*\text{LiCO}_3 + {}^*\text{C} \xrightarrow{Li} {}^*\text{Li}_2\text{CO}_3 + {}^*\text{C}$$

Supplementary Fig.28. Five possible ways and corresponding intermediates on catalysts in Li-CO_2 batteries.

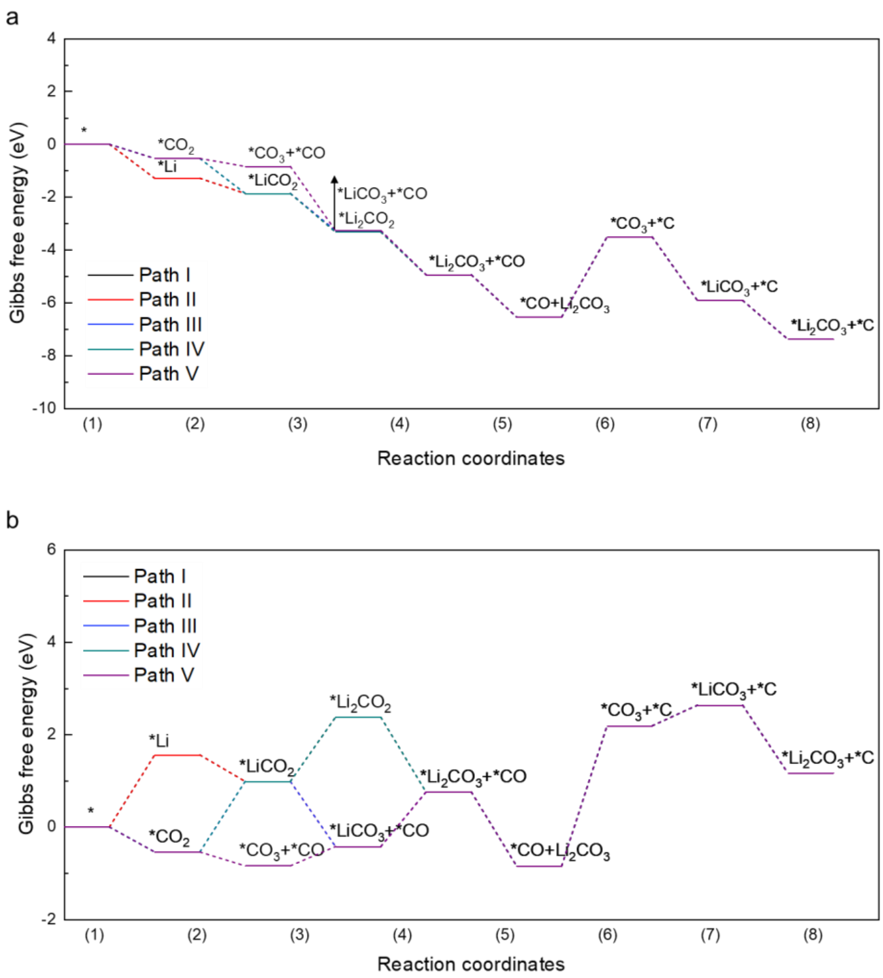

Supplementary Fig.29. Calculated energy profiles on the (311) plane of Co_9S_8. a an open circuit potential (U=0 V) and **b** a theoretical equilibrium potential ($U=U_0$=2.85 V).

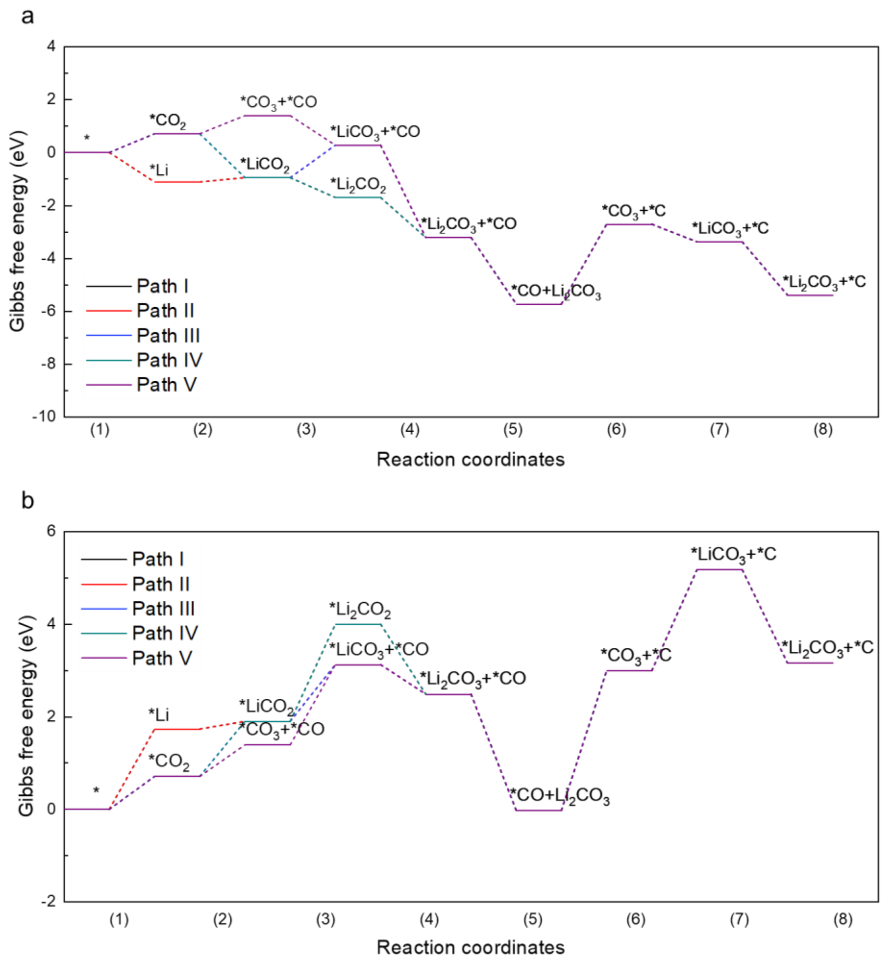

Supplementary Fig.30. Calculated energy profiles on the (204) plane of $CoS_{1.097}$. **a** an open circuit potential (U=0 V) and **b** a theoretical equilibrium potential ($U=U_0=2.85$ V).

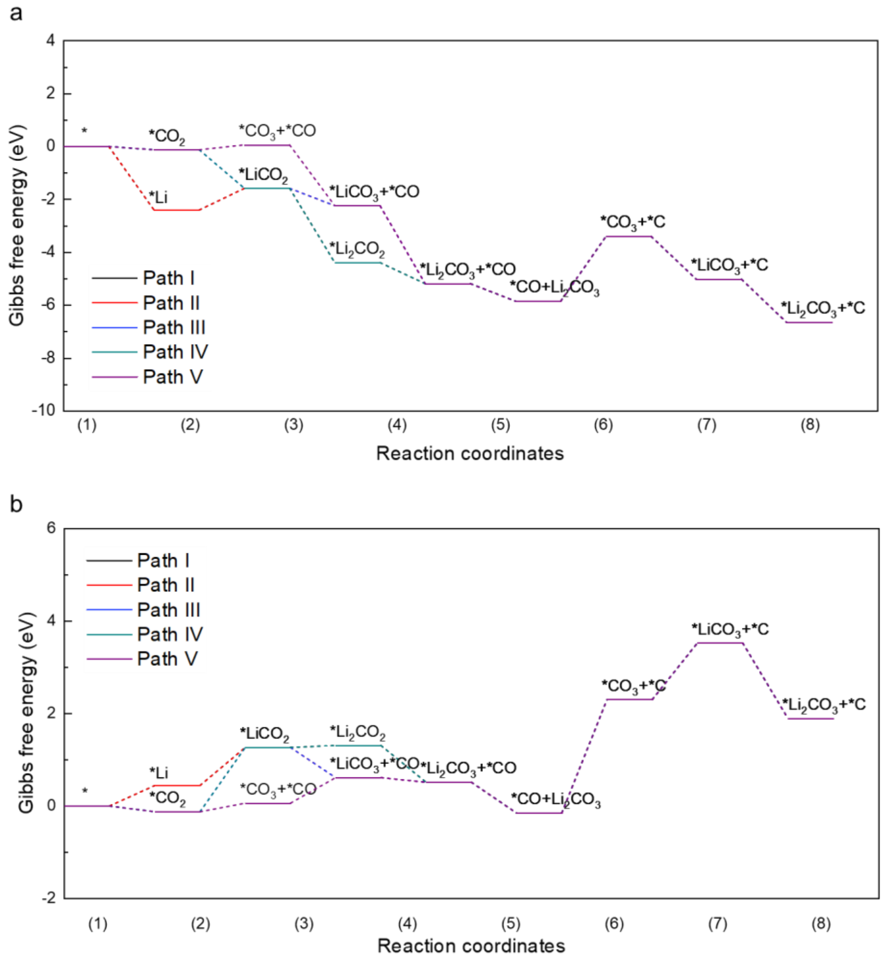

Supplementary Fig.31. Calculated energy profiles on the (200) plane of CoS_2. a an open circuit potential (U=0 V) and **b** a theoretical equilibrium potential ($U=U_0=2.85$ V).

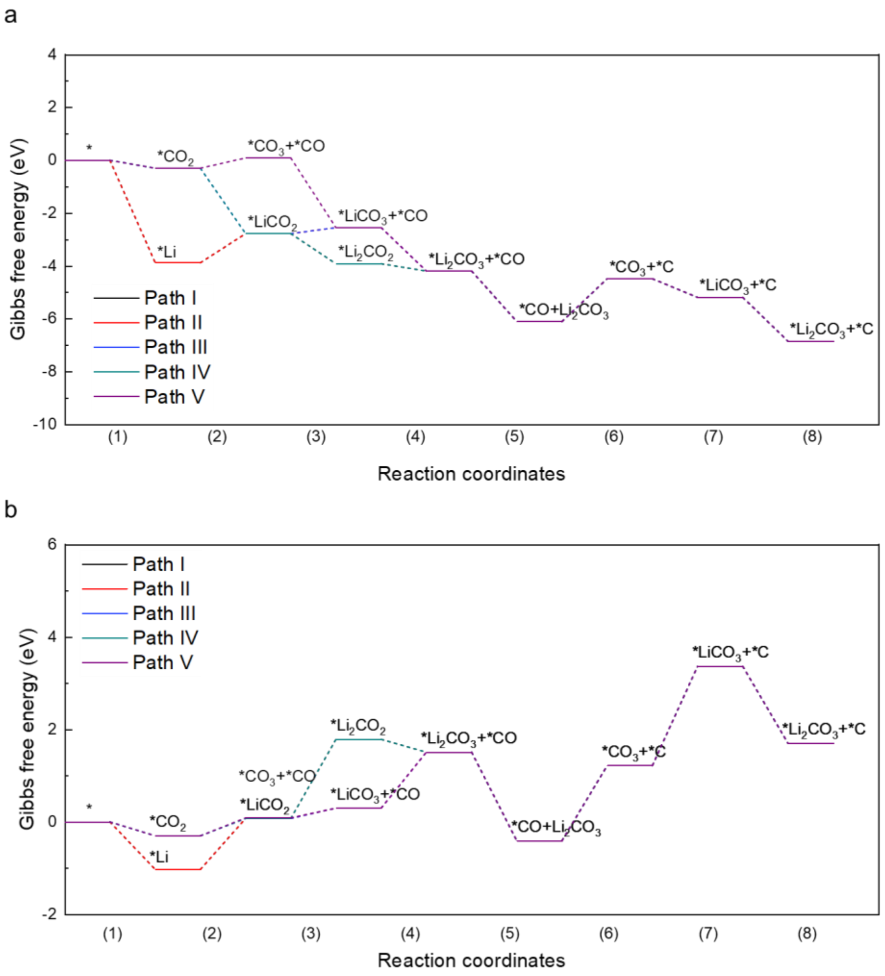

Supplementary Fig.32. Calculated energy profiles on O-CoS_2. a an open circuit potential (U=0 V) and **b** a theoretical equilibrium potential ($U=U_0$=2.85 V).

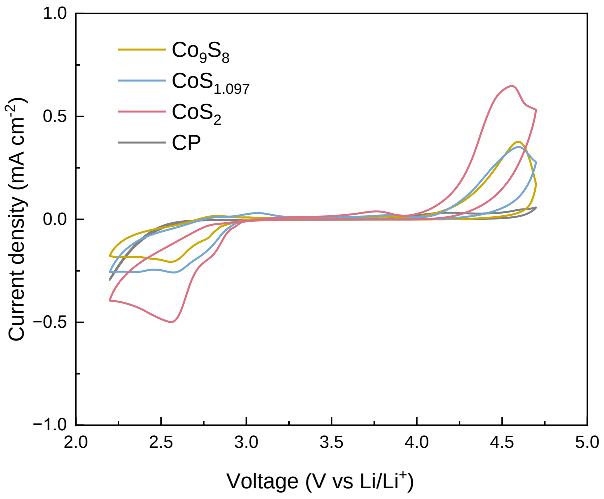

Supplementary Fig.33. CV curves at a scanning rate of 0.1 mV s^{-1} between 2.2 and 4.7 V.

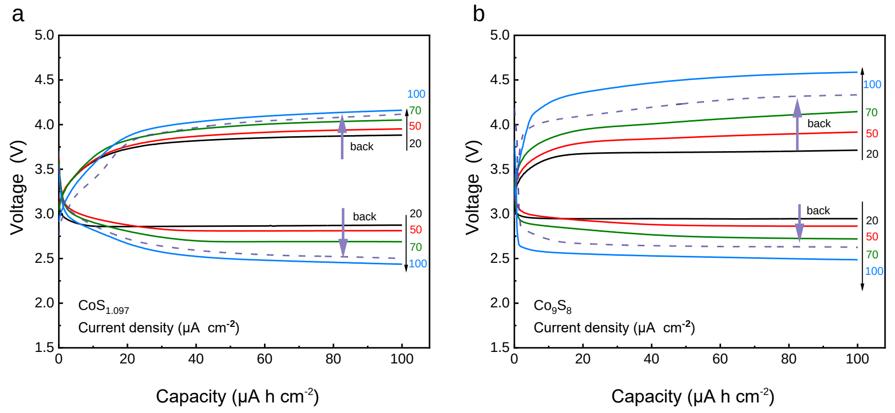

Supplementary Fig.34. GDC profiles with a limited capacity of 100 μA h cm^{-2} at different current densities for Li-CO_2 battery. a $CoS_{1.097}$ and **b** Co_9S_8.

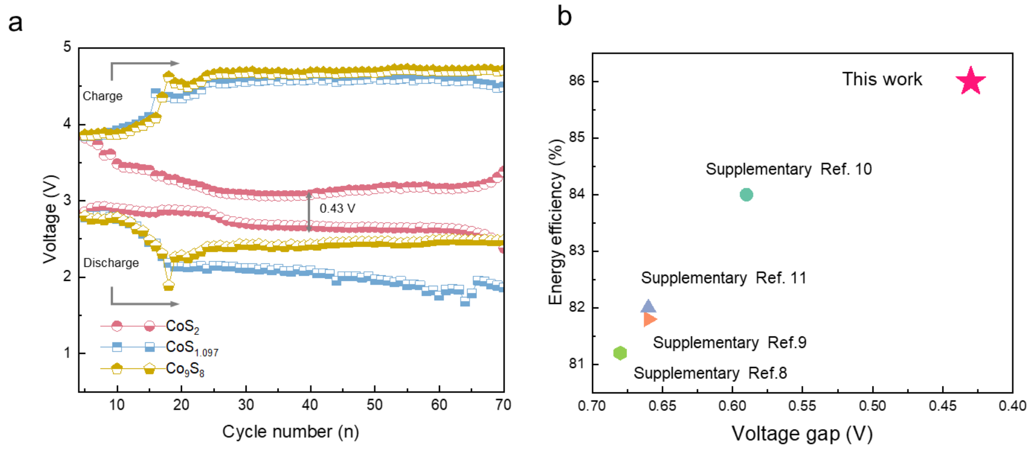

Supplementary Fig.35. Electrochemical performance of CoS_x. a Discharge and charge voltage of long-term cycling for the three cells. **b** Comparison of the voltage gaps and energy efficiency for Li-CO_2 batteries with sulfide cathodes[8, 9, 10, 11].

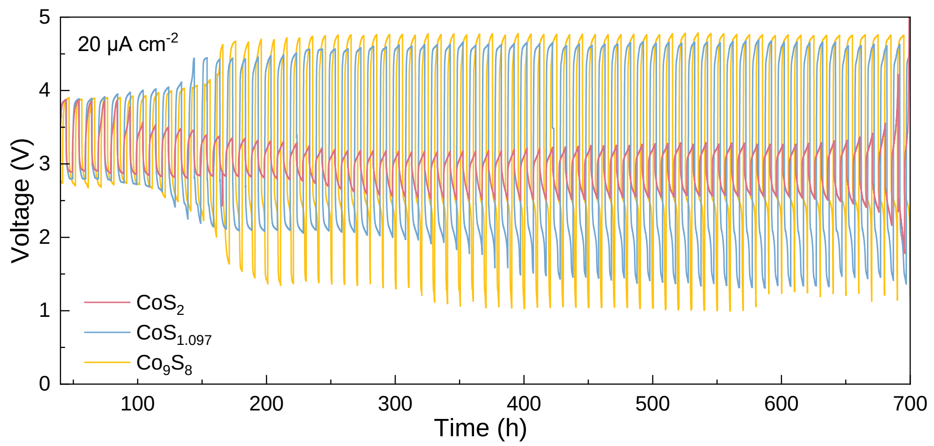

Supplementary Fig.36. Time-voltage curves of long-term cycling for the three cells with a limited capacity of 100 μA h cm^{-2}.

Supplementary Tables

Supplementary Table 1. Comparison of the voltage gaps and energy efficiency for Li-CO_2 batteries. (The discharge products are Li_2CO_3 and C)

Cathode catalysts	Current density	Discharge/ charge voltage (V)	Overpotential(V)	Energy efficiency	Reference
**Carbon-based catalysts**					
Bamboo-like N-doped carbon nanotube fiber	50 mA g^{-1}	2.72/3.98	1.26	75.8%	[12]
N-doped carbon nanotube	50 mA g^{-1}	≈2.73/≈4.24	≈1.51	≈65%	[13]
N-doped CNTs sandwiched between two N-doped graphene layers	100 mA g^{-1}	2.77/3.90	1.13	71%	[14]
N,S-doped CNTs		2.63/4.3	1.67	61.1%	[15]
**Single atom catalysts**					
SACr@NG/PCF	20 μA cm^{-2}	3.1/3.92	0.81	78.7	[16]
Ruthenium atomic cluster and single atom Ru-N_4 composite sites on carbon nanobox substrate	100 mA g^{-1}	3.01/4.06	1.05	74%	[17]
Single Fe atoms of interconnected porous N,S-codoped holey	100 mA g^{-1}	≈2.78/3.95	≈1.17	≈70%	[18]

graphene architecture					
Adjacent Co atoms on graphene oxide	100 mA g^{-1}	≈2.51/≈4.15	≈1.64	≈60%	[19]
**Metal catalysts**					
Iridium nanoparticles embedded in carbon nanofiber networks	100 mA g^{-1}	2.76/4.14	1.38	66.7%	[20]
Ir nanosheets on N-doped, highly porous carbon nanofibers	100 mA g^{-1}	≈2.75/≈3.8	≈1.05	≈72.3%	[21]
Nickel/ruthenium hexagonal nanoplates	200 mA g^{-1}	2.87/3.75	0.88	76.5%	[22]
Ru nanoparticles on N, S co-doped graphene	100 mA g^{-1}	2.91/4.04	1.13	72%	[23]
Ruthenium nanoparticles on carbon nanofibers	100 mA g^{-1}	2.8/4.15	1.35	67.5%	[24]
Ruthenium-copper nanoparticles on carbon nanofibers	100 mA g^{-1}	2.8/3.7	0.9	75.6%	[25]
Ni nanoparticles on the r-GO	200 mA g^{-1}	2.81/4.17	1.36	67.3%	[26]

**Metal oxide catalysts**					
IrO_2 on N doped carbon nanotube	100 mA g^{-1}	2.61/3.95	1.34	66%	[27]
$Co_{0.1}Ni_{0.9}O_x$ nanoparticles on carbon nanotube	100 mA g^{-1}	2.68/4.24	1.56	63.2%	[28]
NiO with oxygen vacancy on carbon cloth	-	2.3/3.5	1.2	66%	[29]
Porous Mn_2O_3	50 mA g^{-1}	≈2.5/≈4.3	≈1.8	58%	[30]
NiO on carbon nanotube	100 mA g^{-1}	≈2.7/≈4.1	≈1.4	65.8%	[31]
α-MnO_2 nanowires	100 mA g^{-1}	≈2.63/≈4.03	≈1.4	65.2%	[32]
**Metal sulfide catalysts**					
ReS_2	20 µA cm^{-2}	2.82/3.47	0.66	81.8%	[9]
CuInS	20 µA cm^{-2}	2.81/3.4	0.59	84%	[10]
MoS_2	50 µA cm^{-2}	≈2.85/3.51	0.66	≈82%	[11]
MoS_2 vertically on Co_9S_8	20 µA cm^{-2}	2.98/3.67	0.68	81.2%	[8]
O-CoS_2 (after 400 cycles)	20 µA cm^{-2}	2.67/3.10	0.43	86%	This work

The overpotential-energy efficiency comparisons are plotted in Fig. 1a. From that we found that carbon catalysts usually have high voltage gap (>1.0 V) and low energy efficiency (<80%). The single atom catalysts based on carbon material have various electrochemical performance, depending on the center atoms and local structures. Metal catalysts usually exhibit better performance than metal oxide catalysts, but mostly are precious metal. Most metal sulfide catalysts have excellent performance with overpotentials around 0.6 V and energy efficiency higher than 80 %.

Supplementary Table 2. Structural parameters of CoS_2 and CoS_2-10 and reference samples that are extracted from the Co *K*-edge EXAFS fitting (S_0^2=0.71).

Sample	Atomic scatter	No. of atoms (CN)	Interatomic distance (Å)	ΔE_0 (eV)	Debye-Waller factor (10^{-3}×Å^2)	R factor
CoS_2	Co-S	6.00	2.27±0.003	-4.29	9.15	0.0095
CoS_2-10	Co-O	1.85±0.28	2.00±0.03	-5.47	10.52	0.0017
	Co-S	3.70±0.56	2.27±0.01	-5.47	5.18	

Note: The background subtraction, merging, normalization, and fitting of the XAS data were performed by Demeter software package.[33] The k^3-weighted EXAFS of Co *K*-edge was Fourier transformed to real (R) space using a Hanning window (dk = 1.0 Å^{-1}) in k-space between 2.398 and 11.150 Å^{-1}. The amplitude-reduction factor S_0^2 was determined by fitting the experimental data of CoS_2. The R-ranges for the fitting of all the EXAFS data were set as 1.0-2.5 Å.

Supplementary Table 3. The measurement value and theoretical amount of CO_2 evolution.

Amount of Li_2CO_3 (μg)	Theoretical amount of Li_2CO_3 (μmol)	Measurement value of CO_2 evolution (μmol)	
		External standard 1#	External standard 2#
525	7.09	6.29	4.28
262.5	3.55	3.20	2.04
131.25	1.77	1.48	0.98

Supplementary Table 4. The quantities of Li_2CO_3 after discharge and charge on the three cathodes.

Cathode	Measurement value of CO_2 evolution (μmol)		Amount of Li_2CO_3 (μmol)		Discharge efficiency of Li_2CO_3 formation	Conversion efficiency of Li_2CO_3
	After discharge	After charge	After discharge	After charge		
Co_9S_8	1.46	1.18	1.68	1.37	89.8%	18.4%
$CoS_{1.097}$	0.91	0.55	1.07	0.67	57.2%	37.4%
CoS_2	1.24	0.61	1.44	0.73	77.0%	48.8%

Discharge efficiency of Li_2CO_3 formation is defined as N_{ad}/N_{th}.

The conversion efficiency of Li_2CO_3 is defined as $(N_{ad}-N_{ac})/N_{ad}$..

N_{ad} is the amount of Li_2CO_3 after discharge, N_{ac} is the amount of Li_2CO_3 after charge, and N_{th} is the theoretical amount of Li_2CO_3 formation after discharge. For the quantitative experiments including in situ DEMS test and titration, the discharge and charge capacity is 100 μA h cm^{-2}, consistent with the electrochemical test. So N_{th} in Supplementary Table 4 is 1.87 μmol for a $2e^-/Li_2CO_3$ process.

Supplementary Table 5. The amount of CO_2 evolution and Li_2CO_3 consumption during charge.

Cathode	Amount of CO_2 evolution (μmol)	Amount of Li_2CO_3 consumption (μmol)	Li_2CO_3/CO_2	e^- /CO_2	e^- /Li_2CO_3
Co_9S_8	0.41	0.31	~0.76	~0.22	~0.17
$CoS_{1.097}$	0.47	0.40	~0.85	~0.25	~0.21
CoS_2	1.24	0.70	~0.56	~0.66	~0.37

The amount of Li_2CO_3 consumption is collected from the titration results in Supplementary Table 4, for which electrodes are extracted from Swagelok cells after discharge and charge with the same electrochemical procedures as in situ tests.

Supplementary Table 6. The adsorption energy (eV) of CO_2, Li and Li_2CO_3 on Co_9S_8, $CoS_{1.097}$, CoS_2 and O-CoS_2.

	**Co_9S_8**	**$CoS_{1.097}$**	**CoS_2**	**O-CoS_2**
**CO_2**	-0.93	0.34	-0.50	-0.66
**Li**	-2.37	-2.21	-3.37	-4.36
**Li_2CO_3**	-1.33	-1.18	-2.46	-3.96

Supplementary Table 7. The number of charge (|e|) of surrounded Co and S/O on CoS_2 and O-CoS_2. The Co sites 1-5 are delineated by dashed cycle in Supplementary Fig.27.

	**CoS_2**	**O-CoS_2**
**Co-1**	8.97	8.67
**Co-2**	8.97	8.67
**Co-3**	8.97	8.96
**Co-4**	9.01	8.86
**Co-5**	8.97	8.96
**S/O**	6.08	6.95

Supplementary Table 8. The Gibbs free energy change of each reaction step on Co_9S_8.

Reaction Step	I	II	III	IV	V
**(1) (U=0 V), ΔG (eV)**	-1.29	-1.29	-0.54	-0.54	-0.54
**(2) (U=0 V), ΔG (eV)**	-0.57	-0.57	-1.32	-1.32	-0.3
**(3) (U=0 V), ΔG (eV)**	-1.46	-1.41	-1.41	-1.46	-2.43
**(4) (U=0 V), ΔG (eV)**	-1.62	-1.67	-1.67	-1.62	-1.67
**(5) (U=0 V), ΔG (eV)**	-1.60	-1.60	-1.60	-1.60	-1.60
**(6) (U=0 V), ΔG (eV)**	3.03	3.03	3.03	3.03	3.03
**(7) (U=0 V), ΔG (eV)**	-2.40	-2.40	-2.40	-2.40	-2.40
**(8) (U=0 V), ΔG (eV)**	-1.47	-1.47	-1.47	-1.47	-1.47
**(1) (U=2.85 V), ΔG (eV)**	**1.56**	**1.56**	-0.54	-0.54	-0.54
**(2) (U=2.85 V), ΔG (eV)**	-0.57	-0.57	**1.52**	**1.52**	-0.30
**(3) (U=2.85 V), ΔG (eV)**	1.39	-1.41	-1.41	1.39	0.42
**(4) (U=2.85 V), ΔG (eV)**	-1.62	1.18	1.18	-1.62	**1.18**
**(5) (U=2.85 V), ΔG (eV)**	-1.60	-1.60	-1.60	-1.60	-1.60
**(6) (U=2.85 V), ΔG (eV)**	**3.03**	**3.03**	**3.03**	**3.03**	**3.03**
**(7) (U=2.85 V), ΔG (eV)**	0.45	0.45	0.45	0.45	0.45
**(8) (U=2.85 V), ΔG (eV)**	-1.47	-1.47	-1.47	-1.47	-1.47

As shown in Supplementary Table 8, the rate determining steps in all pathways I-V are (6) at equilibrium potential on Co_9S_8. For paths V, the Gibbs free energy change of second rate-determining steps are 1.18 eV (step (4)), which is smaller than that of path I and II with the value of 1.56 eV (step (1)) and path III and IV with the value of 1.52 eV (step (2)). Therefore, the path V is the most possible way for the reactions on Co_9S_8.

Supplementary Table 9. The Gibbs free energy change of each reaction step on $CoS_{1.097}$.

**Reaction Step**	**I**	**II**	**III**	**IV**	**V**
**(1) (U=0 V), ΔG (eV)**	-1.11	-1.11	0.71	0.71	0.71
**(2) (U=0 V), ΔG (eV)**	0.16	0.16	-1.66	-1.66	0.68
**(3) (U=0 V), ΔG (eV)**	-0.75	1.23	1.23	-0.75	-1.12
**(4) (U=0 V), ΔG (eV)**	-1.51	-3.49	-3.49	-1.51	-3.49
**(5) (U=0 V), ΔG (eV)**	-2.51	-2.51	-2.51	-2.51	-2.51
**(6) (U=0 V), ΔG (eV)**	3.02	3.02	3.02	3.02	3.02
**(7) (U=0 V), ΔG (eV)**	-0.67	-0.67	-0.67	-0.67	-0.67
**(8) (U=0 V), ΔG (eV)**	-2.01	-2.01	-2.01	-2.01	-2.01
**(1) (U=2.85 V), ΔG (eV)**	1.74	**1.74**	0.71	0.71	0.71
**(2) (U=2.85 V), ΔG (eV)**	0.16	0.16	1.19	1.19	0.68
**(3) (U=2.85 V), ΔG (eV)**	**2.10**	1.23	**1.23**	**2.10**	**1.73**
**(4) (U=2.85 V), ΔG (eV)**	-1.51	-0.64	-0.64	-1.51	-0.64
**(5) (U=2.85 V), ΔG (eV)**	-2.51	-2.51	-2.51	-2.51	-2.51
**(6) (U=2.85 V), ΔG (eV)**	**3.02**	**3.02**	**3.02**	**3.02**	**3.02**
**(7) (U=2.85 V), ΔG (eV)**	**2.18**	**2.18**	**2.18**	**2.18**	**2.18**
**(8) (U=2.85 V), ΔG (eV)**	-2.01	-2.01	-2.01	-2.01	-2.01

As shown in Supplementary Table 9, the rate determining steps and second rate-determining steps in all pathways I-V are (6) and (7) at equilibrium potential on $CoS_{1.097}$. For paths III, the Gibbs free energy change of third rate-determining steps are 1.23 eV (step (4)), which is smaller than that of path I, II, IV and V with the value of 2.10 eV (step (4)), 1.74 eV (step (1)), 2.10 eV (step (4)), 1.73 eV (step (4)). Therefore, the path III is the most possible way for the reactions on $CoS_{1.097}$.

Supplementary Table 10. The Gibbs free energy change of each reaction step on CoS_2.

**Reaction Step**	**I**	**II**	**III**	**IV**	**V**
**(1) (U=0 V), ΔG (eV)**	-2.40	-2.40	-0.12	-0.12	-0.12
**(2) (U=0 V), ΔG (eV)**	0.82	0.82	-1.46	-1.46	0.19
**(3) (U=0 V), ΔG (eV)**	-2.81	-0.65	-0.65	-2.81	-2.30
**(4) (U=0 V), ΔG (eV)**	-0.80	-2.95	-2.95	-0.80	-2.95
**(5) (U=0 V), ΔG (eV)**	-0.67	-0.67	-0.67	-0.67	-0.67
**(6) (U=0 V), ΔG (eV)**	2.46	2.46	2.46	2.46	2.46
**(7) (U=0 V), ΔG (eV)**	-1.62	-1.62	-1.62	-1.62	-1.62
**(8) (U=0 V), ΔG (eV)**	-1.63	-1.63	-1.63	-1.63	-1.63
**(1) (U=2.85 V), ΔG (eV)**	0.45	0.45	-0.12	-0.12	-0.12
**(2) (U=2.85 V), ΔG (eV)**	**0.82**	**0.82**	**1.39**	**1.39**	0.19
**(3) (U=2.85 V), ΔG (eV)**	0.04	-0.65	-0.65	0.04	**0.55**
**(4) (U=2.85 V), ΔG (eV)**	-0.80	-0.10	-0.10	-0.80	-0.10
**(5) (U=2.85 V), ΔG (eV)**	-0.67	-0.67	-0.67	-0.67	-0.67
**(6) (U=2.85 V), ΔG (eV)**	**2.46**	**2.46**	**2.46**	**2.46**	**2.46**
**(7) (U=2.85 V), ΔG (eV)**	**1.23**	**1.23**	**1.23**	**1.23**	**1.23**
**(8) (U=2.85 V), ΔG (eV)**	-1.63	-1.63	-1.63	-1.63	-1.63

As shown in Supplementary Table 10, the rate determining steps and second rate-determining steps in all pathways I-V are (6) and (7) at equilibrium potential on CoS_2. For paths V, the Gibbs free energy change of third rate-determining steps are 0.55 eV (step (3)), which is smaller than that of path I, II, III and IV with the value of 0.82 eV (step (1)), 0.82 eV (step (2)), 1.39 eV (step (2)), 1.39 eV (step (2)). Therefore, the path V is the most possible way for the reactions on CoS_2.

Supplementary Table 11. The Gibbs free energy change of each reaction step on O-CoS_2.

**Reaction Step**	**I**	**II**	**III**	**IV**	**V**
**(1) (U=0 V), ΔG (eV)**	-3.88	-3.88	-0.30	-0.30	-0.30
**(2) (U=0 V), ΔG (eV)**	1.10	1.10	-2.48	-2.48	0.39
**(3) (U=0 V), ΔG (eV)**	-1.14	0.22	0.22	-1.14	-2.64
**(4) (U=0 V), ΔG (eV)**	-0.27	-1.64	-1.64	-0.27	-1.64
**(5) (U=0 V), ΔG (eV)**	-1.92	-1.92	-1.92	-1.92	-1.92
**(6) (U=0 V), ΔG (eV)**	1.63	1.63	1.63	1.63	1.63
**(7) (U=0 V), ΔG (eV)**	-0.71	-0.71	-0.71	-0.71	-0.71
**(8) (U=0 V), ΔG (eV)**	-1.66	-1.66	-1.66	-1.66	-1.66
**(1) (U=2.85 V), ΔG (eV)**	-1.03	-1.03	-0.30	-0.30	-0.30
**(2) (U=2.85 V), ΔG (eV)**	1.10	1.10	0.37	0.37	0.39
**(3) (U=2.85 V), ΔG (eV)**	**1.71**	0.22	0.22	**1.71**	0.21
**(4) (U=2.85 V), ΔG (eV)**	-0.28	**1.21**	**1.21**	-0.28	**1.21**
**(5) (U=2.85 V), ΔG (eV)**	-1.92	-1.92	-1.92	-1.92	-1.92
**(6) (U=2.85 V), ΔG (eV)**	**1.63**	**1.63**	**1.63**	**1.63**	**1.63**
**(7) (U=2.85 V), ΔG (eV)**	**2.14**	**2.14**	**2.14**	**2.14**	**2.14**
**(8) (U=2.85 V), ΔG (eV)**	-1.66	-1.66	-1.66	-1.66	-1.66

As shown in Supplementary Table 11, the rate determining steps and second rate-determining steps in all pathways I-V are (6) and (7) at equilibrium potential on O-CoS_2. For paths II, III and V, the Gibbs free energy change of third rate-determining steps are 1.21 eV (step (4)), which is smaller than that of path I and IV with the value of 1.71 eV (step (3)). The Gibbs free energy change of step (2) are 0.37 eV of path III are smaller than path II and V. Therefore, the path III is the most possible way for the reactions on O-CoS_2

Supplementary References

1. Ouyang C, Wang X, Wang S. Phosphorus-doped CoS2 nanosheet arrays as ultra-efficient electrocatalysts for the hydrogen evolution reaction. *Chem Commun* **51**, 14160-14163 (2015).

2. Faber MS, Dziedzic R, Lukowski MA, Kaiser NS, Ding Q, Jin S. High-Performance Electrocatalysis Using Metallic Cobalt Pyrite (CoS2) Micro- and Nanostructures. *J Am Chem Soc* **136**, 10053-10061 (2014).

3. Uhlig I, Szargan R, Nesbitt HW, Laajalehto K. Surface states and reactivity of pyrite and marcasite. *Appl Surf Sci* **179**, 222-229 (2001).

4. Galtayries A, Grimblot J. Formation and electronic properties of oxide and sulphide films of Co, Ni and Mo studied by XPS. *Journal of Electron Spectroscopy and Related Phenomena* **98-99**, 267-275 (1999).

5. Alstrup I, Chorkendorff I, Candia R, Clausen BS, Topsøe H. A combined X-Ray photoelectron and Mössbauer emission spectroscopy study of the state of cobalt in sulfided, supported, and unsupported Co Mo catalysts. *J Catal* **77**, 397-409 (1982).

6. Zhu L*, et al.* Investigation of CoS2-based thin films as model catalysts for the oxygen reduction reaction. *J Catal* **258**, 235-242 (2008).

7. Rehr JJ, Albers RC. Theoretical approaches to x-ray absorption fine structure. *Reviews of Modern Physics* **72**, 621-654 (2000).

8. Lu B*, et al.* Engineering the interfacial orientation of MoS2/Co9S8 bidirectional catalysts with highly exposed active sites for reversible Li-CO2 batteries. *Proceedings of the National Academy of Sciences* **120**, e2216933120 (2023).

9. Chen B*, et al.* Designing Electrophilic and Nucleophilic Dual Centers in the ReS2 Plane toward Efficient Bifunctional Catalysts for Li-CO2 Batteries. *J Am Chem Soc* **144**, 3106-3116 (2022).

10. Chen L, Zhou J, Zhang J, Qi G, Wang B, Cheng J. Copper Indium Sulfide Enables Li-CO2 Batteries with Boosted Reaction Kinetics and Cycling Stability. *ENERGY & ENVIRONMENTAL MATERIALS* **0**, 1-9 (2022).

11. Pipes R, He J, Bhargav A, Manthiram A. Efficient Li–CO2 Batteries with Molybdenum Disulfide Nanosheets on Carbon Nanotubes as a Catalyst. *ACS Appl Energy Mater* **2**, 8685-8694 (2019).

12. Li X*, et al.* Bamboo-Like Nitrogen-Doped Carbon Nanotube Forests as Durable Metal-

Free Catalysts for Self-Powered Flexible Li-CO2 Batteries. *Adv Mater* **31**, 1903852 (2019).

13. Li Y*, et al.* Highly Surface-Wrinkled and N-Doped CNTs Anchored on Metal Wire: A Novel Fiber-Shaped Cathode toward High-Performance Flexible Li–CO 2 Batteries. *Adv Funct Mater* **29**, (2019).

14. Xiao Y*, et al.* High-Performance Li-CO2 Batteries from Free-Standing, Binder-Free, Bifunctional Three-Dimensional Carbon Catalysts. *ACS Energy Lett* **5**, 916-921 (2020).

15. Song L*, et al.* An ultra-long life, high-performance, flexible Li–CO2 battery based on multifunctional carbon electrocatalysts. *Nano Energy* **71**, 104595 (2020).

16. Liu Y*, et al.* Toward an Understanding of the Reversible Li-CO2 Batteries over Metal–N4-Functionalized Graphene Electrocatalysts. *ACS Nano* **16**, 1523-1532 (2022).

17. Lin J*, et al.* Boosting Energy Efficiency and Stability of Li–CO2 Batteries via Synergy between Ru Atom Clusters and Single-Atom Ru–N4 sites in the Electrocatalyst Cathode. *Adv Mater* **34**, 2200559 (2022).

18. Hu C*, et al.* High-Performance, Long-Life, Rechargeable Li-CO2 Batteries based on a 3D Holey Graphene Cathode Implanted with Single Iron Atoms. *Adv Mater* **32**, 1907436 (2020).

19. Zhang BW*, et al.* Targeted Synergy between Adjacent Co Atoms on Graphene Oxide as an Efficient New Electrocatalyst for Li–CO2 Batteries. *Adv Funct Mater* **29**, 1904206 (2019).

20. Wang C, Zhang Q, Zhang X, Wang XG, Xie Z, Zhou Z. Fabricating Ir/C Nanofiber Networks as Free-Standing Air Cathodes for Rechargeable Li-CO2 Batteries. *Small* **14**, 1800641 (2018).

21. Xing Y*, et al.* Crumpled Ir Nanosheets Fully Covered on Porous Carbon Nanofibers for Long-Life Rechargeable Lithium-CO2 Batteries. *Adv Mater* **30**, 1803124 (2018).

22. Fan L*, et al.* Biaxially Compressive Strain in Ni/Ru Core/Shell Nanoplates Boosts Li–CO2 Batteries. *Adv Mater* **34**, 2204134 (2022).

23. Qiao Y*, et al.* Synergistic effect of bifunctional catalytic sites and defect engineering for high-performance Li–CO2 batteries. *Energy Storage Materials* **27**, 133-139 (2020).

24. Qiao Y*, et al.* Transient, in situ synthesis of ultrafine ruthenium nanoparticles for a high-rate Li–CO2 battery. *Energy Environ Sci* **12**, 1100-1107 (2019).

25. Jin Y, Chen F, Wang J, Johnston RL. Tuning electronic and composition effects in

ruthenium-copper alloy nanoparticles anchored on carbon nanofibers for rechargeable Li-CO2 batteries. *Chem Eng J* **375**, 121978 (2019).

26. Qiao Y*, et al.* 3D-Printed Graphene Oxide Framework with Thermal Shock Synthesized Nanoparticles for Li-CO2Batteries. *Adv Funct Mater* **28**, 1805899 (2018).

27. Wu G*, et al.* Design of ultralong-life Li–CO2 batteries with IrO2 nanoparticles highly dispersed on nitrogen-doped carbon nanotubes. *J Mater Chem A* **8**, 3763-3770 (2020).

28. Xiao X*, et al.* Ultrafine Co-Doped NiO Nanoparticles Decorated on Carbon Nanotubes Improving the Electrochemical Performance and Cycling Stability of Li–CO2 Batteries. *ACS Appl Energy Mater* **4**, 11858-11866 (2021).

29. Zheng R*, et al.* Oxygen vacancy engineering of vertically aligned NiO nanosheets for effective CO2 reduction and capture in Li-CO2 battery. *Electrochimica Acta* **383**, 138359 (2021).

30. Ma W, Lu S, Lei X, Liu X, Ding Y. Porous Mn2O3 cathode for highly durable Li–CO2 batteries. *J Mater Chem A* **6**, 20829-20835 (2018).

31. Zhang X*, et al.* High performance Li–CO2 batteries with NiO–CNT cathodes. *J Mater Chem A* **6**, 2792-2796 (2018).

32. Lei D, Ma S, Lu Y, Liu Q, Li Z. High-Performance Li-CO2 Batteries with α-MnO2/CNT Cathodes. *Journal of Electronic Materials* **48**, 4653-4659 (2019).

33. Newville BRM. ATHENA, ARTEMIS, HEPHAESTUS: data analysis for X-ray absorption spectroscopy using IFEFFIT *Journal of Synchrotron Radiation* **12**, 537-541 (2005).
