# Supplementary material for: Deciphering the contributing motifs of reconstructed cobalt (II) sulfides catalysts in Li-CO2 batteries
Source: Nat Commun. 2024 Mar 9;15:2167. doi: 10.1038/s41467-024-46465-8 (PMC10924882; doi:10.1038/s41467-024-46465-8)
Supplement: Supplementary file 3 — Description of Additional Supplementary Files [file 41467_2024_46465_MOESM3_ESM.pdf]

Description of Additional Supplementary Files:

Supplementary Data 1: Computational Model Data
